# Heat waves characteristics intensification across Indian smart cities

**DOI:** 10.1038/s41598-023-41968-8

**Published:** 2023-09-07

**Authors:** Manish Kumar Goyal, Shivam Singh, Vijay Jain

**Affiliations:** Department of Civil Engineering, Indian Institute of Technology Indore, Indore, India

**Keywords:** Climate change, Projection and prediction

## Abstract

Indian cities have frequently observed intense and severe heat waves for the last few years. It will be primarily due to a significant increase in the variation in heat wave characteristics like duration, frequency, and intensity across the urban regions of India. This study will determine the impact of future climate scenarios like SSP 245 and 585 over the heat wave characteristics. It will present the comparison between heat waves characteristics in the historical time (1981 to 2020) with future projections, i.e., D_1_ (2021–2046), D_2_ (2047–2072), and D_3_ (2073–2098) for different climate scenarios across Indian smart cities. It is observed that the Coastal, Interior Peninsular, and North-Central regions will observe intense and frequent heat waves in the future under SSP 245 and 585 scenarios. A nearly two-fold increase in heat wave' mean duration will be observed in the smart cities of the Interior Peninsular, Coastal, and North Central zones. Thiruvananthapuram city on the west coast has the maximum hazard associated with heat waves among all the smart cities of India under both SSPs. This study assists smart city policymakers in improving the planning and implementation of heat wave adaptation and mitigation plans based on the proposed framework for heat action plans and heat wave characteristics for improving urban health well-being under hot weather extremes in different homogeneous temperature zones.

## Introduction

The urban population exceeded half of the global population in 2019, with 55.70% of the world's population living in urban regions. While in Asia, 57% of the population lives in urban regions, increasing rapidly with time. India has the second-highest population in the world, and due to rapid urbanization, it observes highly dense urban regions^1,2^. It will increase the vulnerability of the urban population to extreme weather events and climate change with the decline in the quality of living among urban regions. The increase in urban development in a haphazard manner will impact the microclimate due to significant environmental changes. These changes induce prominently with expansion in urban coverage through increased built-up area, changes in land use, and high population density. It will increase the vulnerability and health losses due to temperature extremes in all the Indian cities as they have higher population density and economic activities than their surrounding regions^3,4^. Heat waves are one of the extreme temperature events commonly referred to as "silent killers" as their impact is observed to increase later with time on public health, economy, environment, and infrastructure components. Cities observed the excessive death and its associated impacts during heat waves due to its intensification with urban heat island effects. It will increase the risk for rapidly urbanizing and populating cities in developing countries like India. The aftermath effects of heat wave events cause air pollution, water scarcity, drought, flood intensification, etc.^5–8^. As ratified by India, the United Nation's Sustainable Development Goals (SDGs) can be related to minimizing the challenges confronted by the regional urban population to achieve sustainable living in urban regions by 2030^9,10^. Therefore, understanding variations in different heat waves characteristics like duration, frequency, and intensity for different smart cities will be the critical area of the present study.

### Heat waves in India

It is observed that among several regions worldwide, South Asian countries like India and Pakistan is highly susceptible to heat wave due to their intricate landscape, dense population, urbanization, and least resilience against extreme events^11^. A heat wave in 2015 across the Indian subcontinent in South Asia killed 2200 and 2500 individuals in India and Pakistan, respectively. After these events in 2015, Indian National Disaster Management Authority (NDMA) declared heat waves as a disaster at the National level as the heat wave impacted nine Indian states. In 2020, its occurrence increased to twenty-three states of India. Therefore, a heat wave with rising impacts in India generates the interest of policymakers and the scientific community in understanding the variability of the heat wave in India due to climate change and anthropogenic influences. Heat waves adversely affect the regional economy, human mortality, and the surrounding ecosystem^12^. The experience of heat varies from person to person, depending on the place of living. In India, the threshold temperature for heat wave occurrence is 30 °C for hilly regions, 37 °C for coastal regions, and 40 °C for the plain regions. Therefore, there are wide variations in the hot weather experience in northern and southern India. In India, heat waves begin with the sun's transition towards the North and spread of accumulated heat from the deserts of the Northwest region, central plateau, and northern plains. Therefore, Heat waves primarily occur in Northwest India and spread gradually over India's eastwards and southward regions. As prevailing winds in the Northwest region are westerlies to the northwest, there will be no westward development of heat waves. Although, heat wave occurrence secondarily will be due to the presence of favorable conditions in the region^13^. In the Indian region, the favorable conditions for heat wave occurrence depend on four conditions which are: (1) Transference and occurrence of hot, dry air over the region, (2) Lack of moisture in the upper atmosphere due to restriction of moisture through increased temperature, (3) cloudless or clear sky for providing maximum insulation in the region, and (4) Presence of large amplitude anticyclonic flow in the region.

Based on the following reasons, the Indian region observes two types of heat waves; firstly, the North Central India heat wave is associated with blocking over the North Atlantic region, and secondly, associated with East Coastal Region due to the varying Matsuno-Gill response to the abnormal cooling in the Pacific. The heat wave over India has teleconnections with the El Nino Southern Oscillation (ENSO) and variations in the sea surface temperature across the Bay of Bengal^14^. India, in the sub-tropical location, receives ample solar radiation during the summer months, which increases the surface temperature. The presence of deserts and arid regions in northwest and central India leads to the formation of dry air masses, which move towards the Indian subcontinent and intensify heat waves. The stable atmospheric conditions with the absence of convective clouds reduce the precipitation and prevail the hot waves in the region. The greenhouse gas emissions and urban built-up intensify the greenhouse and urban heat island effect in the cities and change the heat wave characteristics in the region^15,16^. The climate oscillation, like the negative phase of the Indian Ocean dipole (IOD) and North Atlantic variability, intensifies the dryness and warmer condition in the Indian region. The Pacific meridional mode (PMM), which is the combination of the coupled ocean and atmospheric variability in the North-Eastern Pacific observed to weaken the walker circulation in the Pacific Ocean, which intensifies the easterlies in the region and amplifies the hot weather conditions in the Indian subcontinent. Among all the conditions for heat wave occurrence, it is observed the shortwave radiation fluctuations in the region are the significant contributing factor to the intensification of the heat wave characteristics in the region^17–20^. It adversely impacts the Indian urban population's health, infrastructure development, water resources, energy consumption, transport services, etc. Urban health and well-being due to heat waves are impacted by a wide range of factors differently across homogeneous temperature zones. In the plain and coastal regions, we observed more intense urban heat island effects, vulnerable populations, and mortality due to heat waves than in hilly regions of India. In hilly regions, heat wave impacts less in the valley and shaded regions than in exposed slope and higher elevations. In coastal regions, the sea breeze and humidity are prominent factors in determining the impact of heat waves. The lesser efficient planning and implementation of the heat action plan by policy makers in several states caused significant mortality in different parts of India in different years, including in 2023. For example, Uttar Pradesh and Bihar state governments observed several lapses in heat action plans in 2023 due to the mortality of hundred individuals in the north-central region^21^. As per the Indian Meteorological Department, heat waves in India are prominent mainly in pre-monsoon conditions from April to June^22,23^. In the Indian context, as per IMD, a heat wave called for a period of at least three or more consecutive days^13^. Therefore, this study will assess the heat wave's characteristics like duration, frequency, and intensity during April, May, and June, both for historical and forthcoming years under different climate scenarios and propose a framework for better implementation of heat action plan at the city level to reduce the risk of hot weather extremes across urban regions of India.

### CMIP 6 assessment

The historical heat wave datasets were determined through Indian Meteorological Department (IMD), while futuristic heat wave datasets were evaluated through Coupled Model Intercomparison Projections. The Coupled Model Intercomparison Project (CMIP) initiated in 1995 by the World Climate Research Program (WCRP) is in 6th phase. It will give a better understanding of future heat wave predictions using CMIP 6 projections. Recently, several researchers have studied futuristic changes in heat waves worldwide by applying CMIP 6 climate projections. The CMIP 6 projections comprised five high priority Shared Socioeconomic Pathways Scenarios (SSPs). These five SSPs were classified as sustainability, middle-of-the-road development, regional rivalry, inequality, and fossil-fueled development. These are used to assess emissions scenarios considering climate change or mitigation scenarios. These emission scenarios will be used to determine climate change projections. SSPs assist climate change analysts in preparing climate policy^24–27^. This study considers SSP 245, i.e., Middle Road development, and SSP 585, i.e., fossil-fueled development. In SSP 245, the world follows a development pathway according to the historical, socioeconomic, and technological development in the twenty-first century. In SSP 585, the world becomes more competitive, innovative and has highly participatory groups to produce swift technological advancement and expansion of human capital as the path to sustainable development. It will consider maximum mitigation challenges and minimum adaptation measures^24,28,29^. The CMIP 6 projections assessment for different climate scenarios frequently assessed by several researchers over India to assess past and future trends for heat waves like Bhattacharya et al.^30^, Das et al.^31^, Das and Umamahesh^22^, Hari et al.^17^, Nandi and Swain^32^, Tokarska et al.^33^. These future perspectives through CMIP 6 assessment were significantly correlated with Indian urban regions, making them disaster resilient to hot weather extreme weather events.

### Why Indian smart cities?

Presently, 31% of India's people reside in cities, contributing to 63% of the nation's GDP. By 2030, 40% of India's population will be urban residents, contributing 75% to the nation's GDP. It is estimated that more than 50% of India's population will be urbanized by 2050^34^. Thus, urban regions must overcome several challenges of climate change and extreme weather events to enhance the nation's development and economy. Keeping these ideas in perspective, The Government of India, in 2015, selected 100 Indian smart cities to make them climate-resilient and centers of development and economic progress^35^. In September of the same year, 2015, India ratified the 2030 Plan of Sustainable Development Goals (SDGs). Presently, India has ranked 121 among the 163 countries worldwide among global ranking for SDGs (NITI Aayog, 2022). One of these goals is SDG 11, which refers to "Make Cities and Human Settlements Safe, Inclusive, Resilient and Sustainable." India has declining performance in SDG 11 due to factors like quality of life, disaster resilience, declining air quality, water stress conditions, etc^36,37^. Heat waves are a prominent extreme weather phenomenon in almost all Indian cities. It impacts the urban population by causing a loss in work productivity through physical and mental stress, water scarcity, air pollution, etc. Urbanization creates large impervious surfaces that influence local surface morphology, meteorological and hydrological conditions. Therefore, this study will be the first of its kind to assess the quantification of heat wave characteristics both for historical and forthcoming years under different climate scenarios for better adaptation and implementation of mitigation measures to minimize the risk associated with heat waves by identification of the hazard associated with heat waves and increase the well-being of urban residents under hot weather extremes with the following objectives:

- Heat Waves Characteristics Historical Assessment for the Indian Smart Cities
- Heat Waves Characteristics Future Assessment under CMIP 6 Projections for SSP 245 and SSP 585 scenarios among Indian Smart Cities
- Heat Waves hazard assessment for the Indian smart cities and proposing the framework for the heat wave action plan at the city level

**Figure 1:**
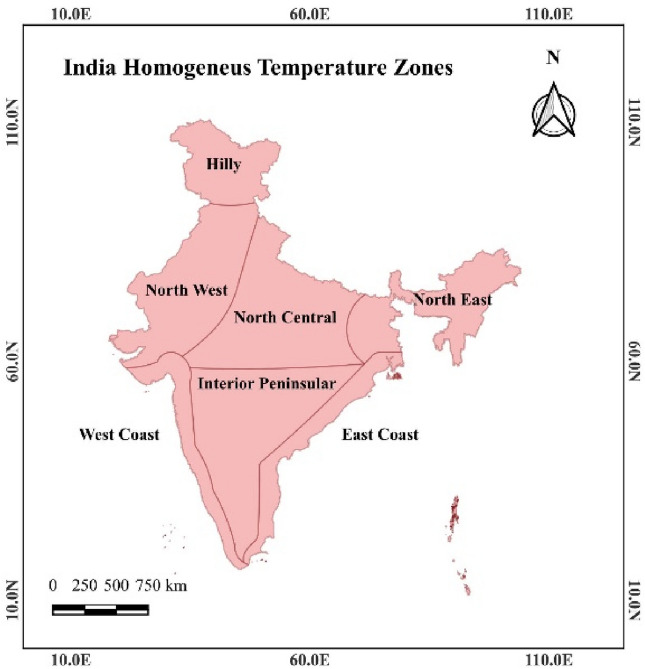

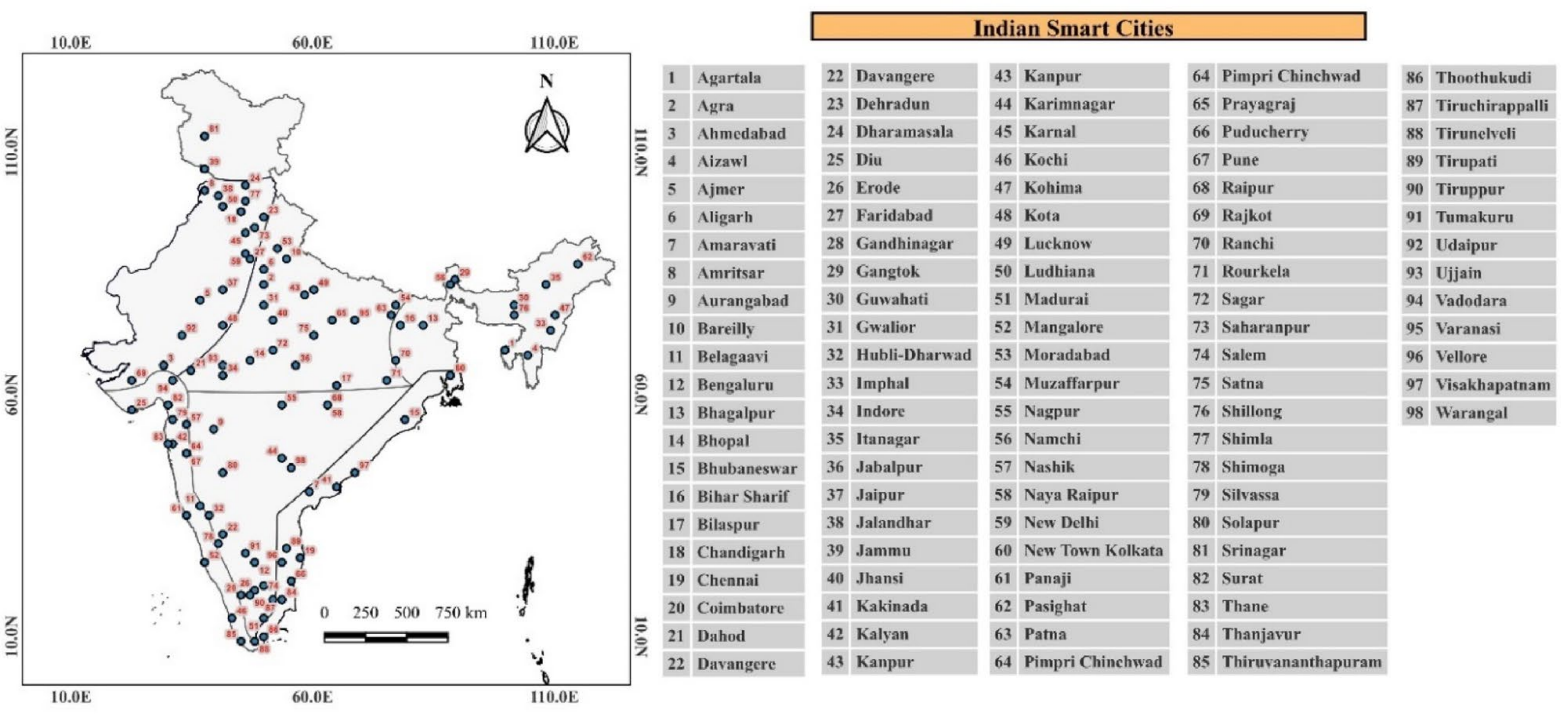
(**a**) Seven homogeneous temperature zones across India^40^. (**b**) Location map of the Indian smart cities across homogeneous temperature zones^40^.

Heat wave dominate India's urban regions due to the urban heat island effect, high anthropogenic influence, and climate change^38,39^. Among all the hundred Indian smart cities, Port Blair and Kavaratti cities were not selected in the study region due to the non-availability of specific temperature datasets over these urban regions. Therefore, this study will provide insights into SSP 245 and SSP 585 CMIP 6 future projections of heat waves in comparison with historical datasets for Indian smart cities with comparative assessment for northern and southern Indian cities across seven homogeneous temperature zones of India, as shown in Fig. 1a,b respectively.

## Results and discussions

The heat wave assessment for the Indian smart cities is classified and presented across seven homogeneous temperature zones. This section presents the duration, frequency, and intensity of the heat wave characteristics from April to June for Indian smart cities in historical and future periods through SSP 245 and SSP 585 projections.

### Duration

This study considers heat wave duration to be at least three consecutive days. Heat wave duration is the mean consecutive days for sustained heat waves over the region. Therefore, heat wave duration indicates the mean consecutive days among 91 days from April to June (AMJ). The heat wave events mean duration between April to June across Indian smart cities presented in detail based on the homogeneous temperature zones below:

### Hilly and northeast zones

These zones have fifteen cities with the least occurrence of heat wave events in historical time compared to other zones. The mean duration of heat waves in smart cities of these zones, SSP 245 (4.5D) and 585 (8.5D) increased by 46.7% and 100%, respectively. The maximum heat wave mean duration during historical SSP 245 and 585 scenarios was observed in the Guwahati (24 days), Bihar Sharif (35 days), and Gangtok (41 days) cities, respectively. The Aizwal and Imphal cities in this region have not observed any heat wave events in historical times. Overall, the increase in heat wave events mean duration during both SSPs maximum in Bihar Sharif, Bhagalpur, and Jammu cities.

### North central zone

This region has twenty-two cities surrounded by Northwest, Northeast, and Interior Peninsular zones. The mean duration of the heat wave in SSP 245 (4.5D) and 585 (8.5D) increased by 74.8% and 139%, respectively. The maximum heat wave duration during historical SSP 245 and 585 scenarios was observed in the Rourkela (23 and 40 days) and Jhansi (48 days) cities, respectively. Overall, the increase in heat wave events mean duration during both SSPs maximum in Varanasi, Kanpur, and Lucknow cities. The heat wave duration occurrence is nearly one month since the D2 period under both SSPs, with a maximum in the month of June.

### Northwest zone

This region has nineteen cities surrounded by Northcentral, Hilly, and West Coast zones. The mean duration of the heat wave in SSP 245 (4.5D) and 585 (8.5D) increased by 64.6% and 126.6%, respectively. The maximum heat wave duration during historical SSP 245 and 585 scenarios was observed in the cities of Ajmer (20 days) and Dharamshala (32 and 40 days). Overall, the increase in heat wave events mean duration during both SSPs maximum in Saharanpur, Dehradun, and Faridabad cities. The heat wave duration occurrence is one month since the D2 period under both SSPs, with a maximum in the month of June.

### Interior peninsular zone

This region has nineteen smart cities surrounded by coastal and north-central regions. The mean duration of heat waves in smart cities in SSP 245 (4.5D) and 585 (8.5D) increased by 132.4% and 268.1%, respectively. The maximum heat wave duration during historical SSP 245 and 585 scenarios was observed in Solapur (19 days) and Tirunelveli city (45 and 60 days), respectively, as shown in Fig. 6. Coimbatore and Tumakuru cities in this region have not observed any heat wave events in historical time. Overall, the increase in heat wave events mean duration during both SSPs maximum in Tirupur, Bengaluru, and Erode cities. The heat wave duration occurrence is nearly one month since the D_2_ period under both SSPs, with a maximum during the month of May.

### East and west coastal zones

**Figure 2:**
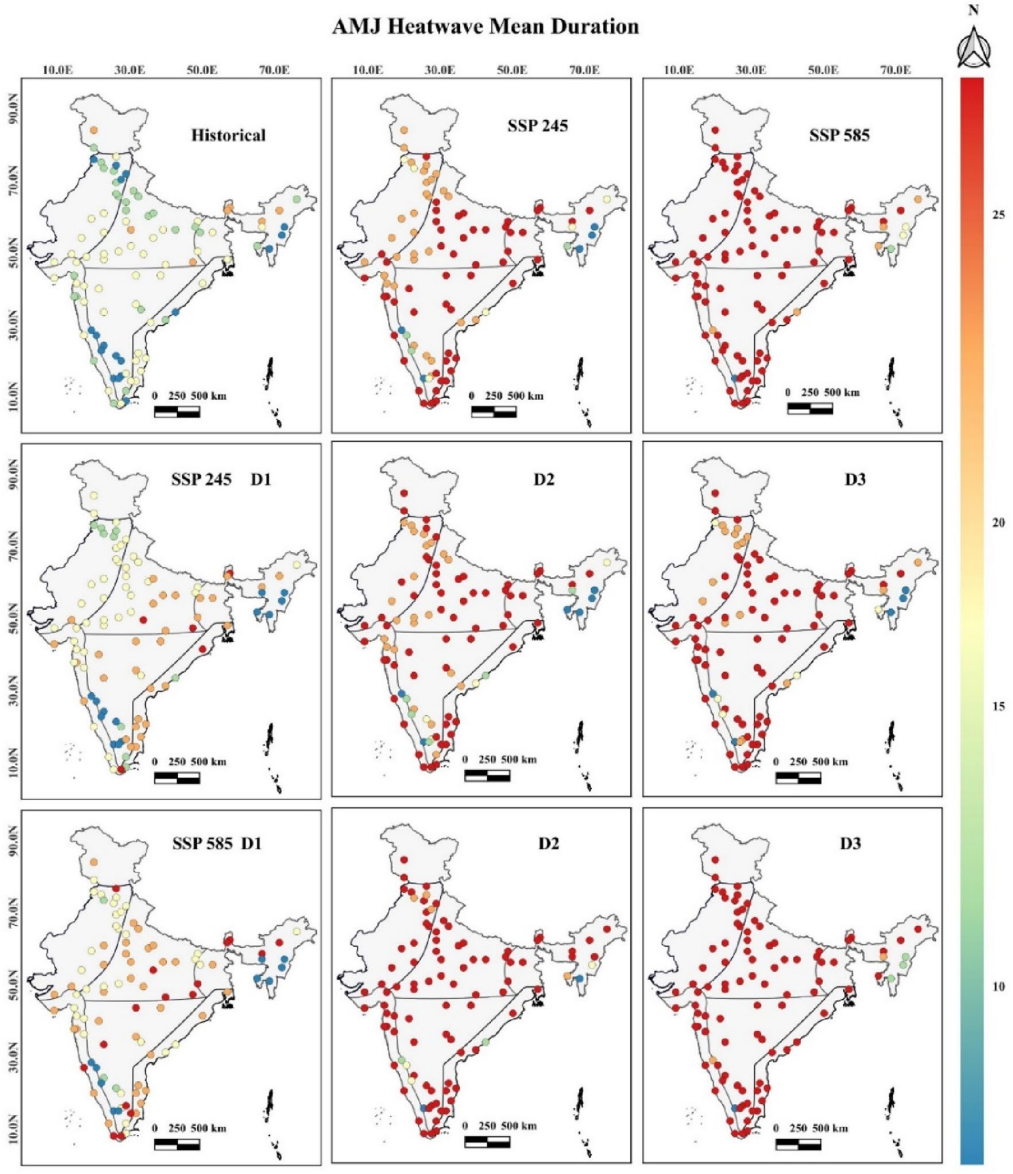
Heat wave mean duration between April to June^40^.

**Figure 3:**
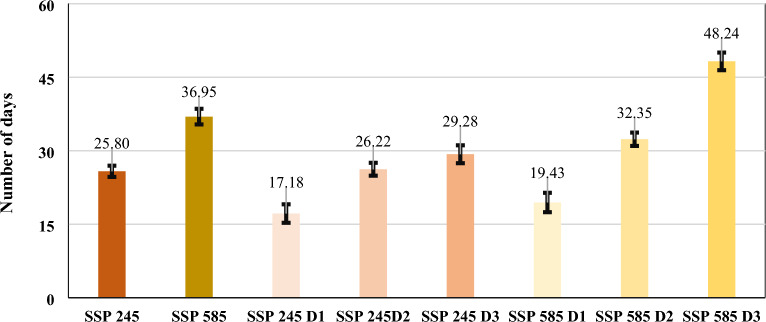
Heat wave mean duration with 95% confidence intervals for SSP 245 and 585 scenarios.

The coastal region has ten and thirteen smart cities on India's east and west coasts. The mean duration of heat waves in smart cities of these zones increases in SSP 245 (4.5D) and 585 (8.5D) by 86.6% and 173.3%, respectively. The maximum heat wave average duration during historical SSP 245 and 585 scenarios was observed in the Thanjavur (19 days) and Thiruvananthapuram (47 and 62 days) cities, respectively, as shown in Fig. 2. Belagavi city in these regions has not observed any heat wave events in historical times. Overall, the increase in heat wave events mean duration during both SSPs maximum in Thoothukudi, Mangalore, and Thiruvananthapuram cities. The heat wave's mean duration occurrence is nearly one month since the D2 period under both SSPs, with a maximum during the month of May. The heat waves mean duration, shows some common trends, which are as follows: (a) Historically, heat wave duration is observed maximum of 15 to 20 days, (b) In SSP 245, the maximum duration will be observed with more than 30 days since the Second Decadal period, and (c) In SSP 585, the maximum duration will be observed with more than 60 days since the Second Decadal period. The heat wave mean duration values with 95% confidence intervals considering all the smart cities in different temperature zones have been shown for SSP 245 and 585 climate scenarios in Fig. 3.

### Frequency

The frequency of heat waves is defined as the count of the mean number of sub-heat waves that occurred in the region between April to June. As per IMD, the sub-heat wave has a spell of at least three consecutive days. Thus, the frequency of the heat wave is directly proportional to the duration of the heat wave due to the criteria of three consecutive hot days. Due to the proportionality between heat wave duration and frequency, their spatial extent is similar, with varying magnitude across Indian smart cities. In the Hilly and Northeast Zones of India, the heat wave events mean frequency under SSP245 and 585 will increase by 35.85% and 62.17%, respectively. The maximum heat wave frequency during historical SSP 245 and 585 scenarios was observed in Itanagar city.

**Figure 4:**
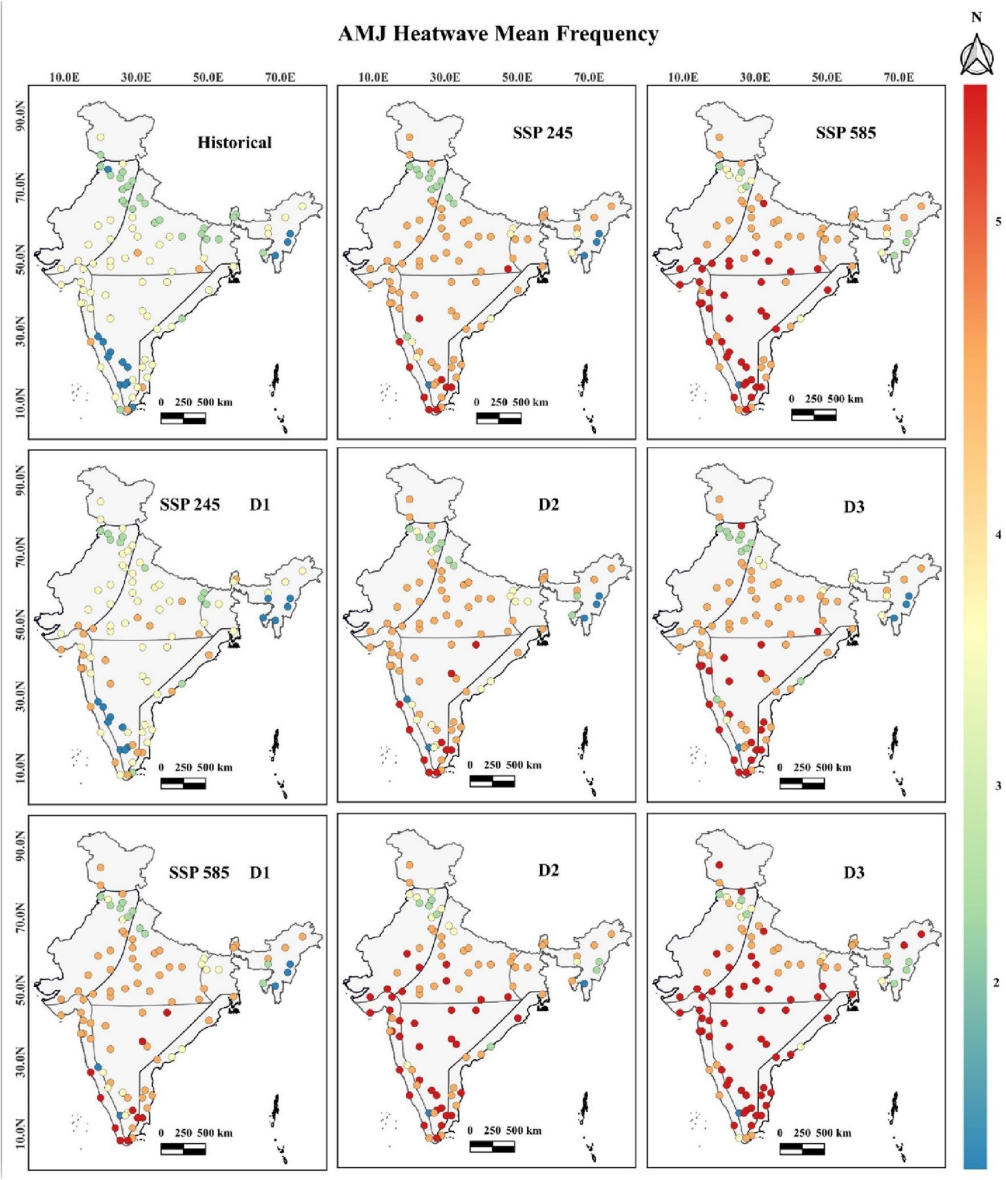
Heat wave mean frequency between April to June^40^.

**Figure 5:**
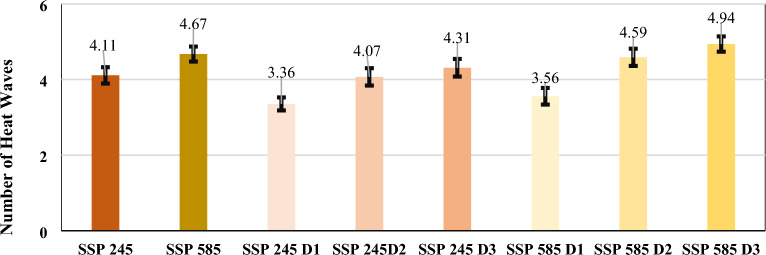
Heat wave mean frequency numbers with 95% confidence intervals for SSP 245 and 585 scenarios.

The coastal region of India observed an increase in the mean frequency of heat wave events in SSP 245 and 585 by 37.87% and 49.48%, respectively. Historically, the most frequent heat waves were observed for Thanjavur city across the east coast. In the future, under both SSPs, the maximum frequency was observed for the Panaji, and Pimpri Chinchwad across the west coast cities, respectively. The Interior peninsular region mean frequency under both SSPs increased by 79.09% and 107.21%, respectively. Maximum frequent heat waves in historical and SSP 245 time periods were observed in Tirunelveli city, while in SSP 585, it will be observed across Hubli-Dharwad city. The northcentral zone observed an increase in heat wave frequency under both SSPs by 30.59% and 40.82%, respectively. Heat wave are most frequent in Rourkela during historical and SSP 245 time, while in SSP 585, it will be maximum for Bareilly city. The Northwest zone observed an increased heat wave frequency under both SSPs by 31.89% and 50%, respectively. Heat waves are most frequently observed in Ajmer, Dharamshala, and Ahmedabad during both historical and SSPs. In SSP 245, during the decadal period, the frequency of D2 and D3 heat wave events showed a maximum increase across Interior Peninsular, East, and West coastal cities, respectively. While in SSP 585, an abrupt increase in frequent heat waves was observed in the North-central, Interior Peninsular, East, and West coastal cities with certain parts of Northwest, Hilly, and Northeast zones. Therefore, frequent heat waves are prominently observed under both SSPs across Interior Peninsular, East, and West coastal cities, as shown in Fig. 4 with the following common trends like: (a) Historically, heat wave frequency is observed maximum of 3 to 4 numbers, (b) In SSP 245, the maximum frequency will be observed with more than five numbers since the Third Decadal period, and (c) In SSP 585, the maximum frequency will be observed with more than five numbers since the Second Decadal period. The heat wave mean frequency values with 95% confidence intervals considering all the smart cities in different temperature zones have been shown for SSP 245 and 585 climate scenarios in Fig. 5.

### Intensity

Heat wave intensity is the maximum temperature sustained in urban regions during hot weather events. Intensity is used to assess the region's severity of heat wave events. The heat wave events mean intensity between April to June across Indian smart cities is presented in detail based on the homogeneous temperature zones below:

### Hilly and northeast zones

The heat wave intensity over this region during SSP 245 and 585 will increase by 30.26% and 44.49%, respectively. The intense heat wave during the historical period and both SSPs will be significantly observed in Pasighat (T_max_ > 43 °C) and across the Eastern Himalayan regions of Arunachal Pradesh. The maximum increase in heat wave intensity will be observed in Shillong, Bhagalpur, and Bihar Sharif cities. The mean intensity over this region under SSP 245 and 585 will be more than 30 °C since D_3_ and D_2_ periods across all the cities, with a maximum in the month of June.

### North central zone

The heat wave intensity over this region during SSP 245 and 585 will increase by 11.48% and 17.95%, respectively. During the historical and SSPs, intense heat waves will be significantly observed in Jhansi (T_max_ > 43 °C) and across India's Uttar Pradesh and Madhya Pradesh smart cities. A maximum increase in heat wave intensity under both SSPs will be observed in Ranchi, Patna, and Lucknow cities. The mean intensity over this region under SSP 245 and 585 will be more than 40 °C since the D_2_ period across all the cities, with the maximum in the month of June.

### Northwest zone

**Figure 6:**
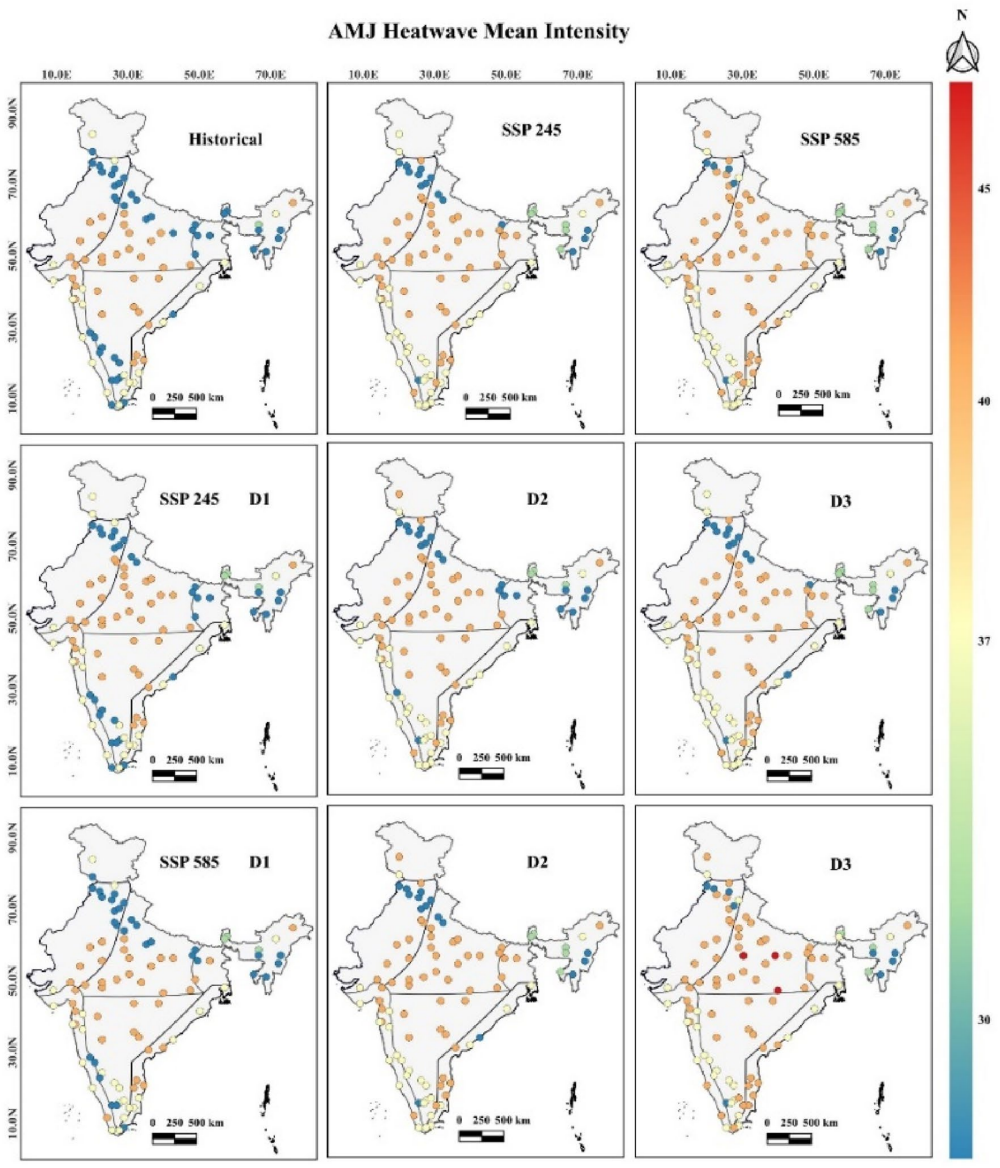
Heat wave mean intensity between April to June^40^.

The heat wave intensity over this region during SSP 245 and 585 will increase by 5.03% and 14.24%, respectively. During the historical and SSPs, intense heat waves will be significantly observed in Ajmer (T_max_ > 43 °C) and across India's Rajasthan and Gujarat smart cities. A maximum increase in heat wave intensity under both SSPs will be observed in New Delhi and Faridabad cities. The mean intensity over this region under SSP 245 and 585 will be more than 36 °C since the D_2_ period across all the cities, with a maximum in the month of June. The variation in the intensity magnitude for all the smart cities under different decadal periods is shown in Fig. 6.

### Interior peninsular zone

The heat wave intensity over this region during SSP 245 and 585 will increase by 35.32% and 33.77%, respectively. During the historical and SSPs, intense heat waves will be significantly observed in Raipur (T_max_ > 43 °C) and across India's Maharashtra and Telangana smart cities. A maximum increase in heat wave intensity under both SSPs will be observed in Tirupur, Erode, and Bengaluru cities. The mean intensity over this region under SSP 245 and 585 will be more than 38 °C since the D_3_ and D_2_ periods across all the cities with a maximum in the month of May.

### East and west coastal zones

**Figure 7:**
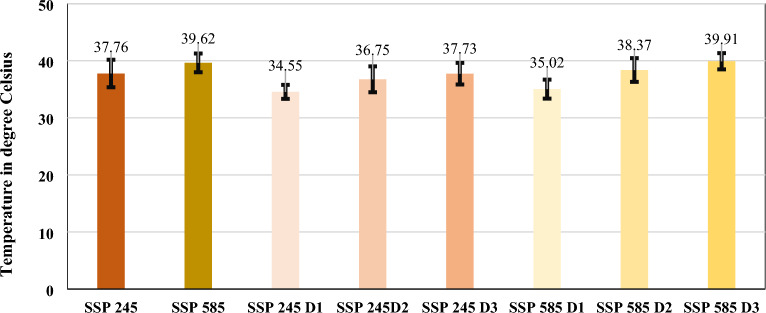
Heat Wave mean intensity with 95% confidence intervals for SSP 245 and 585 scenarios.

The heat wave intensity over this region during SSP 245 and 585 will increase by 10.15% and 11.23%, respectively. Intense heat waves during the historical and both SSPs will be significantly observed in Vadodara (T_max_ > 42 °C) and across many Eastern Coastal smart cities of India. A maximum increase in heat wave intensity under both SSPs will be observed in Thiruvananthapuram, Thoothukudi, and Visakhapatnam. The mean intensity over this region under SSP 245 and 585 will be more than 40 °C since the D_2_ period across all the cities, with a maximum in the month of June. The heat waves mean intensity for all the smart cities shows the following common trends as shown in Fig. 7: (a) Historically, heat wave intensity is observed maximum between 40 to 43 °C, (b) In SSP 245, the maximum mean intensity will be observed to increase by 1 °C, and (c) In SSP 585, the maximum mean intensity will be observed to increase by 2 °C across all the smart cities. The heat wave mean intensity values with 95% confidence intervals considering all the smart cities in different temperature zones have been shown for SSP 245 and 585 climate scenarios in Fig. 7.

Based on the assessment of variation in heat wave characteristics among all the smart cities, we have determined the hazard index for all the cities for historical and future periods. The datasets of mortality due to heat waves are present at the state level used for assessing the vulnerable population due to heat waves are prominent in which temperature zones of India.

### Heat wave hazard index

The heat wave hazard index under the historical SSP 245 and 585 scenarios have been determined individually for all the smart cities. As frequency are the sub-spells of the heat wave to compute duration, we determined the heat wave hazard index for the smart cities based on the projected intensification in mean duration and intensity compared to the threshold maximum temperature. Guwahati and Itanagar in the Hilly and Northeast region have a high hazard historically for heat wave events. Although, in future scenarios, the hazard shows a slight decline in cities of this region. The coastal cities of India show moderate hazards in historical periods for heat wave events. In future scenarios, cities across the west coast will be more vulnerable to heat waves than the East Coast. Thiruvananthapuram city on the west coast has the maximum hazard among all the smart cities of India under both SSPs. In the Interior Peninsular region, heat wave events have a moderate hazard in both historical and future scenarios. Although, cities in Tamil Nādu and Telangana states have increasing hazard trends in the future scenarios. The coimbatore city has the least heat wave hazard in this region among India's smart cities. Ajmer and Kota cities in the Northwest zones have a high hazard historically.

In contrast, the future hazard associated with heat waves may slightly decline in this region compared to other cities in India. The cities of Rajasthan and Gujarat have a high hazard for heat wave events in this region. The North Central zones' cities have the maximum hazard associated with heat wave events under historical and future scenarios.

In this zone, Rourkela, Jhansi, Sagar, Bhopal, Satna, and Gwalior have the maximum hazard historically among all the cities. Madhya Pradesh cities were highly vulnerable to heat wave events in historical and future scenarios.

**Figure 8:**
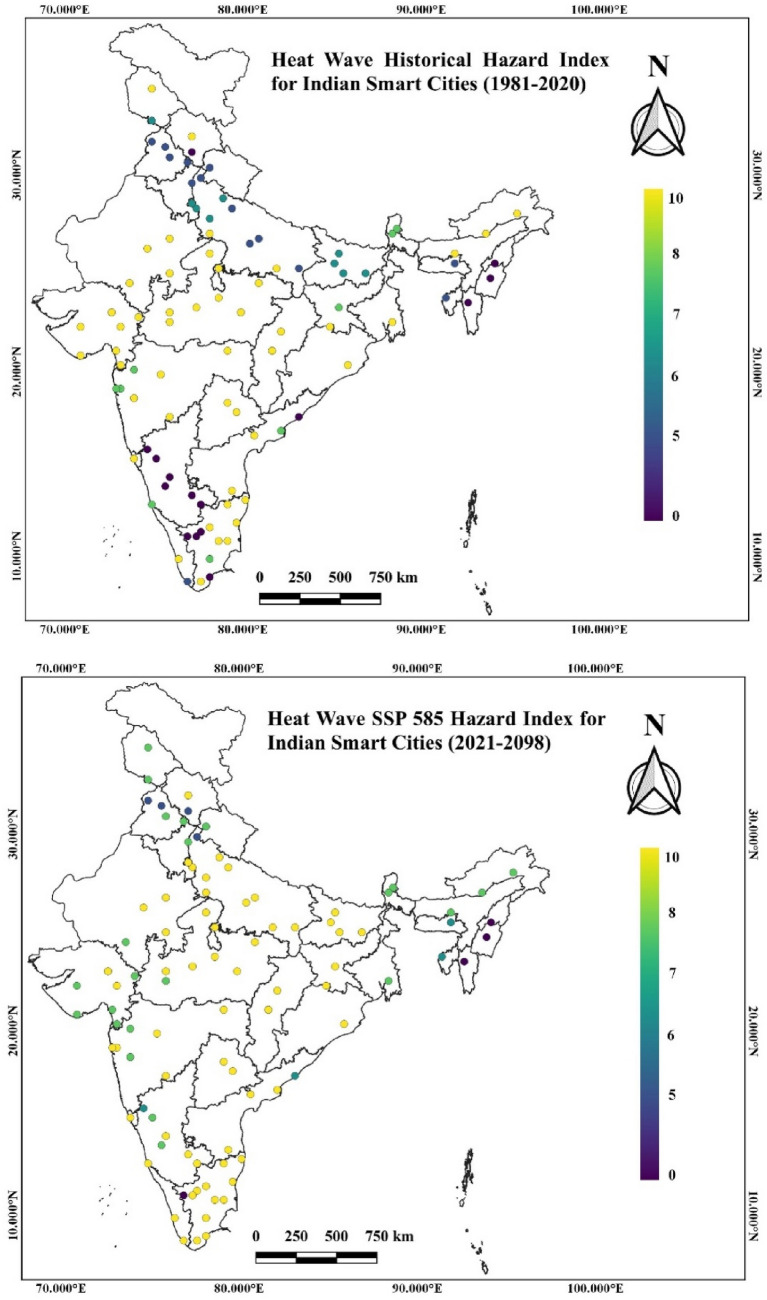
(**a**) Heat waves historical hazard index for Indian smart cities^40^. (**b**) Heat waves SSP 585 hazard index for Indian smart cities^40^.

Figure 8a,b show that heat wave events are significantly higher under SSP 585 for Coastal, Interior Peninsular, and North Central zones cities among all the homogeneous temperature zones. Therefore, by embracing sustainable urban planning, green infrastructure, and public health initiatives, cities can better prepare to respond to the challenges posed by heat waves in a changing climate. The smart cities mission policymakers should adopt the following changes in heat wave characteristics to make these cities resilient to hot weather extremes.

### Heat wave mortality

**Figure 9:**
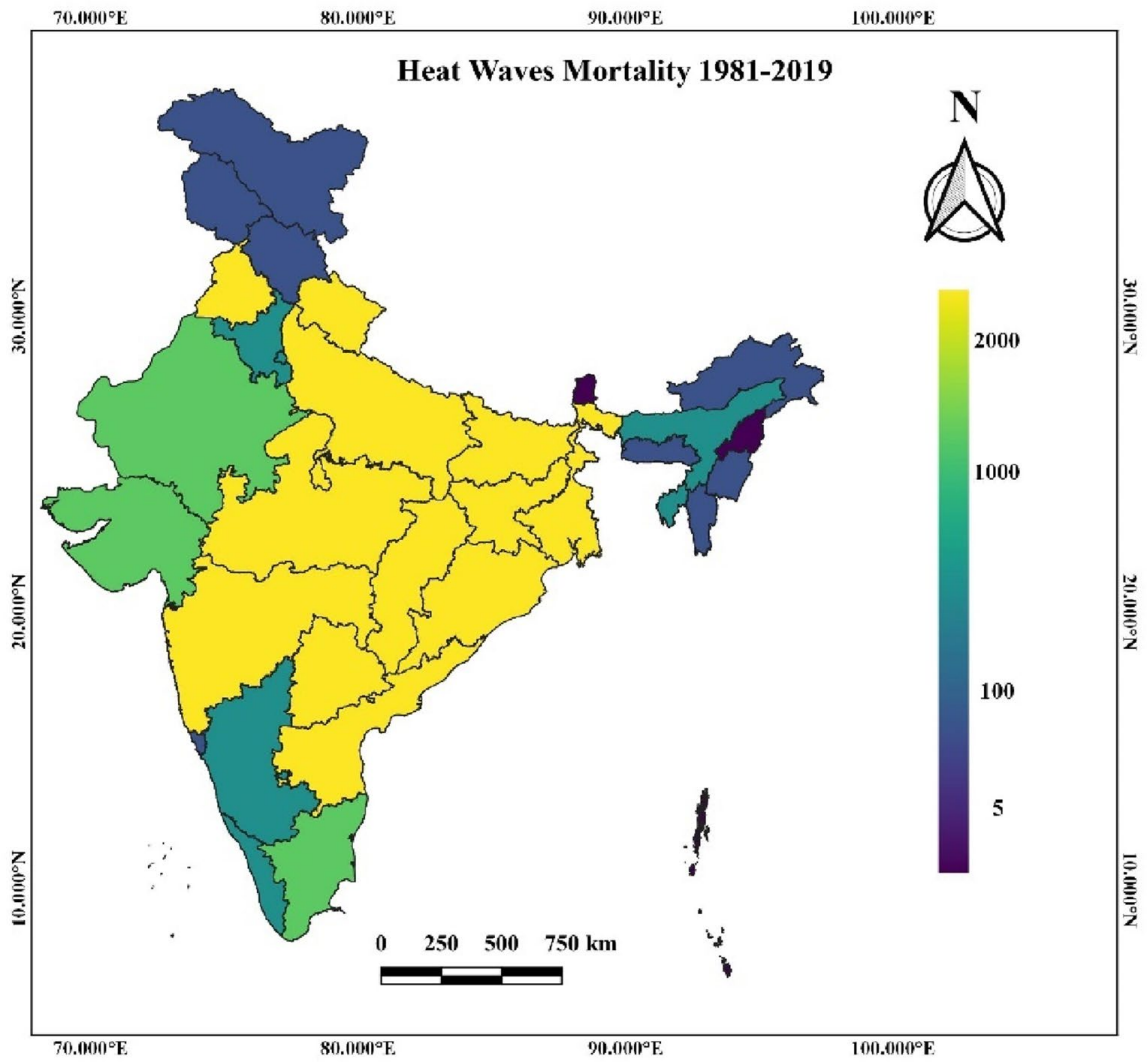
Heat wave mortality across India^40^.

Heat waves are one of the high-risk natural hazards but receive less attention as the mortality and impact associated with them are not always instantly noticeable. Several deaths occurred historically due to heat waves in India annually. However, mortality due to heat waves depends on several regional parameters like humidity, socioeconomic factors, air quality, etc.^13,41,42^. The Mortality datasets due to heat waves are present at the state level from 1981 to 2019. The highest morality is due to the heat wave observed in the North Central Region of India and certain parts of the east coast, as shown in Fig. 9. High population density and fewer adaptation plans for heat waves cause large numbers of mortality in the region. India's Extreme maximum temperature and heat wave are observed in the Northwest region of India, which comprises several deserts like Thar desert in Rajasthan, Rann of Kutch in Gujarat, etc. Although, due to previous adaptation and mitigation plans for heat waves by the local government and population acclimatization helped them reduce the region's mortality. The eastern coastal region of India experiences more mortality due to heat waves than the western coastal region. The lesser coping capacity of the regional population in the east coast region makes them more highly vulnerable to heat wave events than the west coast. In the Interior peninsular region, mortality due to the heat wave was prominently observed in Tamil Nādu, Telangana, and certain parts of Andhra Pradesh and Karnataka. It is probably due to increased population and water stress conditions, rapid urbanization, and extreme temperatures. Hilly and Northeast regions of India have the most negligible mortality due to the slightest observance and hazard of heat waves. The decadal variability among heat waves from 1981 to 1999 and 2000 to 2019 showed a 53.86% increase in mortality rates per million for the Indian population. Arunachal Pradesh, Haryana, Punjab, Andhra Pradesh, and Odisha observed a maximum rise in heat wave mortality from 2000 to 2019.

### Framework for the city-level heatwave action plan

The city-level heat wave action plan should focus on the following components: considering all the regional heat wave drivers, characteristics, hazards associated with hot weather extremes and identifying the vulnerable population and infrastructure. The heat wave action plan should be distributed for at least three phases, i.e., pre-summer, summer, and post-summer. The actions under these phases should be decided primarily by involving all the concerned urban stakeholders and policy makers to consider more regional inputs on assessing the hazard associated with the heat wave in the region for better organization of regional actions and for protecting public health. The heat wave hazard can be assessed through heat wave drivers in the region based on the outcomes of the study. The hot weather drivers at the city level are primarily decided based on three parameters: climate change, urban population growth, and the region's infrastructure development.

Based on regional topography like hilly, plain, and coastal regions, several parameters will be considered for forecasting heat wave events in the region along with maximum temperature in the region like humidity, sunlight exposure to slope in hilly regions, ocean temperatures in coastal regions, etc. The vulnerability during hot weather events in the city will be decided based on the population's exposure to hot weather days, infrastructure type, vulnerable population (like infants, elderly, and people on medications, etc.), and the effectiveness of the response system in case someone impacted by heat waves in the region. Considering hazards associated with the heat waves in the region and vulnerability, the regional policymakers may prepare an adaption and mitigation plan for heat waves based on regional socioeconomic conditions proposed modifications in the urban planning like construction material, infrastructure type, how to reach to the vulnerable populations through early warnings and increasing the regional population capacity building for such events.

Urban planning should focus on increasing the blue and green infrastructure in the region, which plays a major role in minimizing the urban heat island effect in the region. Considering the hazard, vulnerability, and exposure associated with heat waves in the region, we follow the action plan based on its three phases. Under each phase of the action plan, we have to consider two types of action, i.e., short and longer-term development and planning. In the pre-summer period, the short-term development will involve assessing the risk zones in the city, capacity building of the regional population, preparation for early forecasting, with deciding the role and responsibilities of all the concerned stakeholders in the city.

**Figure 10:**
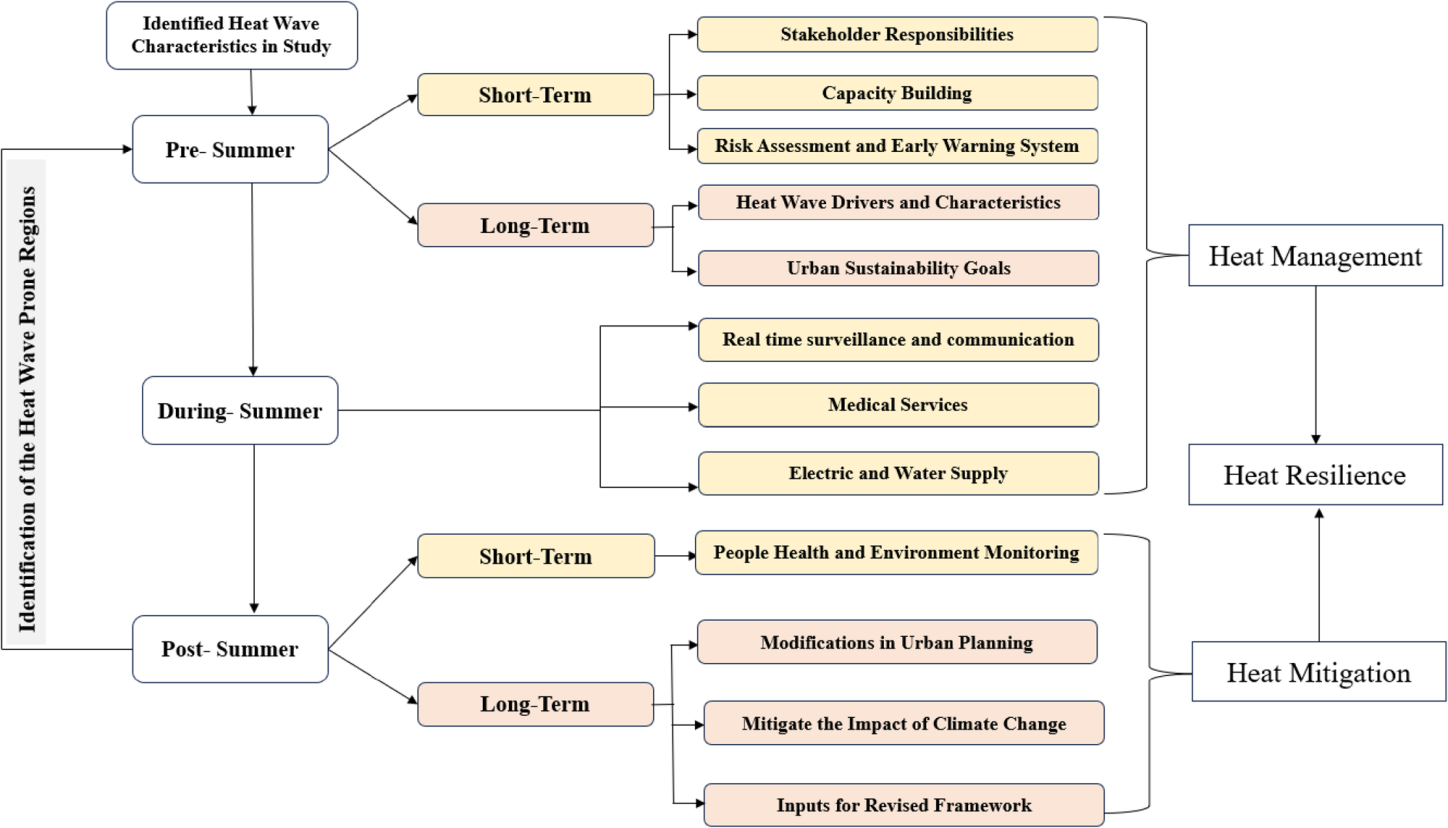
Framework for city-level heat wave action plan.

The long-term planning will include assessing several factors contributing to heat wave occurrence in the region and planning for urban sustainability in the region. During heat wave events, the planning includes providing real-time, efficient alerts through effective communication, prompt medical aid availability, and proper electrical and water supply functioning. The post-summer short-term planning will be focused on the assessment of the health and environment monitoring, while long-term planning includes modifications in urban planning to reduce the risk of heat waves, revisions in the frameworks of the action plan through meeting with concerned stakeholders, and actions to mitigate the impact of climate change by providing urban health well-being through resilience to hot weather events in the region. The complete framework for the heat wave action plan is shown in Fig. 10.

## Conclusions

This study is the first to present heat wave events characteristics assessment for Indian smart cities. It considers natural and anthropogenic causes for the increase in temperature and heat wave occurrences using CMIP 6 projections. The significant outcome of our study will include the identification of the historical trends in the characteristics of the heat waves and its comparison with forthcoming years under two different climate scenarios, i.e., sustainable and fossil fuel development pathways through SSP 245 and 585 scenarios, respectively. Based on the following consideration, we determined the heat wave hazard index for Indian smart cities through heat wave characteristics. We proposed a heat wave action plan framework with strategies for adapting and mitigating hot weather extremes and making cities resilient to climate change and extreme weather events. It will be used to minimize institutional and implementation gaps for the present heat waves action plan at the city level for addressing the issues of land use changes and urban heat island effects. Therefore, considering the transport, energy, and infrastructure sectors, a heat wave adaptation and mitigation action plan must be modified for the high-hazard urban population among all Indian smart cities with the involvement of all the city stakeholders and policy makers.

An efficient heat action plan contributes significantly to regional population health to minimize the risk of climate change or sensitive diseases and make comprehensive socioeconomic policy changes to mitigate and increase urban population adaption to heat waves in India. The cities across temperature zones of Coastal, Interior Peninsular, and North Central regions will observe severe intensification in the heat wave characteristics in the future years. It is observed that the mean duration across these regions by the end of this century in the third decadal period (2073–2098) under the SSP 245 and 585 scenario will be nearly thirty and sixty days, respectively. However, in the North Central region, it will be near to fifty days for SSP 585 scenarios. The frequency of heat waves will increase by more than five numbers in a three-month period which is historically observed with two numbers in maximum cities. The mean intensity was increased by more than five degrees Celsius, with the maximum in the North Central zone, where it is observed to increase by nearly forty-two and forty-four degrees Celsius in both the SSPs, respectively. It will significantly impact day-to-day energy demand, water supply, working hours, and the overall economy across these regions for longer periods.

West coastal smart cities in southern India will observe significantly longer duration, more frequent, and hotter heat waves in the future. Thiruvananthapuram city on the west coast will observe more than sixty days of heat wave duration between April to June every year in the future. The city will observe more than five heat waves with mean increases in intensity by one and two degrees Celsius, respectively, in SSP 245 and 585 scenarios. The west coast region, including Thiruvananthapuram city, observe severe heat wave prominently due to more anticyclonic flow in the Arabian Sea, which influence wind pattern, impact sea breeze, cause subsidence of warm air, and increase the temperature in the region. Apart from these, several other factors, like the rise in the Arabian Sea temperature, affected coastal mainland temperature and the decline in the summer rainfall, cumulatively increasing the maximum temperature in the region. The present framework for the heat wave action plan provide consideration for heat wave risk in the high-risk prone regions like Thiruvananthapuram by computing historical heat wave characteristics and specified consideration for mitigation the impact of climate change based on future projections in the heat wave characteristics as mentioned in the study. It will provide consideration for the urban sustainability goals in the pre-summer period for achieving regional sustainability for enhancing the electric, water supplies and the medical services during the heat wave period in the region.

The present research study focuses on Indian smart cities. However, a similar study can be extended for other cities and regions across India based on the bias correction and correlation of the maximum temperature datasets with different GCMs. The future works in this study will involve considering the humidity and urban heat island effect together with the maximum temperature to provide an inclusive understanding for assessing the hot weather extremes characteristics variations at the city level. Further works to determine the risk associated with the heat wave at the city level should involve identifying the vulnerable population, infrastructure and determining the impact on the city economy due to loss in working hours and mortality in the region. The better identification of risk considering all the parameters like maximum temperature, humidity, and urban heat island effect, along with consideration of vulnerable and exposure parameters, will assist all the urban stakeholders and policymakers in making every city resilient to hot weather extremes in India. Therefore, this research work will be useful for urban policymakers and the scientific community for designing "climate-smart" cities in India under the Indian smart cities mission. It will assist us in achieving the objectives of the Sendai Framework for Disaster Risk Reduction and Sustainable Development Goals (SDGs), specifically for SDG 11, by 2030.

## Datasets and methodology

**Figure 11:**
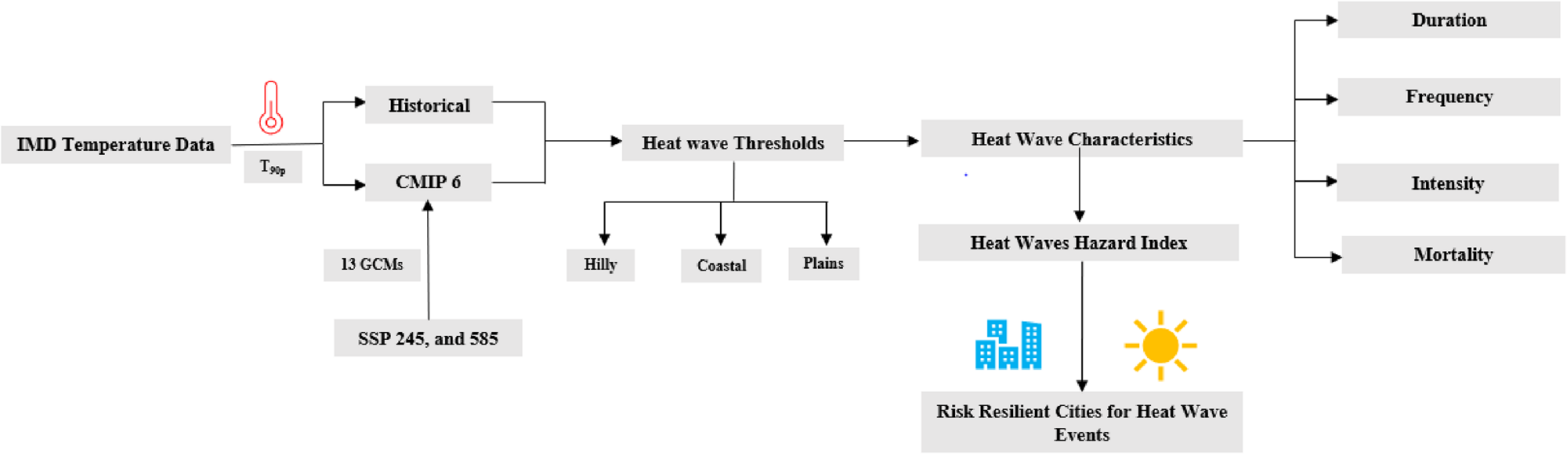
Flow chart for methodology adopted to evaluate heat wave characteristics assessment.

Indian Meteorological Department (IMD), in association with the Indian Institute of Tropical Meteorology (IITM), has identified seven homogeneous temperature zones for India. These zones were classified as Hilly, Northeast, Northwest, North Central, Interior Peninsula, West, and East Coast regions, as shown in Fig. 1.^43^ Among these zones, Hilly and Northeast, Northwest, and North Central are classified as Northern Indian smart cities, while Interior Peninsula, West, and East Coast zones are classified as Southern India smart cities. Thus, to provide a better spatial presentation of a heat wave for the Indian smart cities across homogeneous temperature zones, IMD maximum temperature gridded datasets at 1° * 1° resolution across 350 stations in India was used as a reference dataset in this study^44^. This study assessed the heat wave hazards in Indian smart cities based on the IMD daily maximum temperature datasets observed in April, May, and June from 1981 to 2020. These IMD datasets can be downloaded from https://www.imdpune.gov.in/lrfindex.php. While future temperature projections were derived from CMIP6 projections, which were available through the following link https://zenodo.org/record/3987736#.YHWeiugzaUk. The SSP 245 and SS585 future projections of heat waves in India were evaluated through bias-corrected CMIP 6 maximum temperature datasets at 0.25° * 0.25° spatial resolution produced by Mishra et al.^45^. These bias-corrected maximum temperature datasets were created using thirteen CMIP 6 models for the South Asia region^22,45^. The detailed assessment and statistical investigations for developing this data are described in the study of Mishra et al.^45^. This study used thirteen Global Climate Models (GCMs) to assess CMIP 6 future projections from 2021 to 2098 under SSP 245 and 585 scenarios. A detailed description of the GCMs used in this study is presented in Table 1. The threshold maximum temperature conditions are maximum of either based on analyzing the 90th percentile through the baseline period from 1950 to 2010 or considering the IMD threshold values, i.e., 40 °C for the plains, 37 °C for the coast, and 30 °C over the Hilly regions for more than three days respectively. Based on the threshold temperature and considering the three consecutive days periods, we have determined heat wave parameters, i.e., duration, frequency, and intensity for historical and future periods across Indian smart cities in seven homogeneous temperature zones. The comparison between heat wave parameters among historical time (1981 to 2020) with future projections i.e., D_1_ (2021–2046), D_2_ (2047–2072), and D_3_ (2073–2098) in SSP 245 and 585 presented in this study. It will use to evaluate the increase in intensity and severity of heat wave across Indian smart cities with time. Heat wave severity across homogeneous temperature regions was determined based on mortality associated with heat wave^23^. Heat wave mortality datasets for the Indian region obtained at the state level in the absence of the city level data to assess the vulnerable population for heat wave events in the Indian region. The mortality datasets associated with heat waves between 1981 to 2019 were obtained from reports of the National Crime Records Bureau (NCRB) through the following link https://ncrb.gov.in/en/accidental-deaths-suicides-in-india^42^. Population datasets for this study were obtained from the Government of India 2011 Census data through the link: https://censusindia.gov.in/census.website/hi/data/population-finder. Therefore, based on the duration, frequency, and intensity, we determined the heat wave hazard index for the Indian smart cities. The flow chart for the overall methodology adopted in this study is shown in Fig. 11.

**Table 1:** List of GCMs under CMIP6 projections used in the present study.

Model name	Modeling centre
ACCESS-CM2	CSIRO, Australian Research Council Centre of Excellence for Climate System Science (CSIRO‐ ARCCSS)
ACCESS‐ESM 1‐5	Commonwealth Scientific and Industrial Research Organization, Australia (CSIRO)
BCC‐CSM2‐MR	Beijing Climate Center, China Meteorological Administration, China (BCC)
CanESM5	Canadian Centre for Climate Modelling and Analysis, Canada (CCCma)
EC-Earth3	EC‐EARTH consortium (EC‐EARTH)
EC-Earth3-Veg	EC‐EARTH consortium (EC‐EARTH)
INM‐CM4‐8	Institute for Numerical Mathematics (INM), Russia
INM‐CM5‐0	Institute for Numerical Mathematics (INM), Russia
MPI‐ESM 1‐2‐HR	Max Planck Institute for Meteorology, Deutscher Wetterdienst, Deutsches Klimarechenzentrum, Germany (MPI‐M, DWD, DKRZ)
MPI‐ESM 1‐2‐LR	Max Planck Institute for Meteorology, Alfred Wegener Institute, Germany (MPI‐M, AWI)
MRI‐ESM 2‐0	Meteorological Research Institute, Japan (MRI)
NorESM2‐LM	Norwegian Climate Centre, Norway (NCC)
NorESM2‐MM	Norwegian Climate Centre, Norway (NCC)

## Data availability

All the datasets computed and assessed during the research study will be accessible from the corresponding author on request.
